# Feeling safe: a critical look at the effect of neighborhood safety features and perceptions on childhood symptoms of depression

**DOI:** 10.1186/s12887-024-05236-6

**Published:** 2024-11-27

**Authors:** Erika Infantino, Tracie A. Barnett, Carolyn Côté-Lussier, Andraea Van Hulst, Mélanie Henderson, Marie-Eve Mathieu, Catherine Sabiston, Lisa Kakinami

**Affiliations:** 1 Department of Psychology, Concordia University, Montréal, Québec Canada; 2 Centre de Recherche Azrieli du CHU Sainte-Justine, Montréal, Québec Canada; 3 Department of Family Medicine, McGill University, Montréal, Québec Canada; 4 Institut National de la Recherche Scientifique, Centre Urbanisation Culture Société, Montréal, Québec Canada; 5 Ingram School of Nursing, McGill University, Montréal, Québec Canada; 6 Department of Pediatrics, Université de Montréal, Montréal, Québec Canada; 7 School of Public Health, Department of Social and Preventive Medicine, Université de Montréal, Montreal, Québec Canada; 8 School of Kinesiology and Physical Activity Sciences, Université de Montréal, Montréal, Québec Canada; 9 Faculty of Kinesiology and Physical Education, University of Toronto, Toronto, Ontario Canada; 10 School of Health, Concordia University, Montréal, Québec Canada; 11 Department of Mathematics and Statistics, Concordia University, 1455 de Maisonneuve West, Montréal, Québec Canada; 12 Science College, Concordia University, Montréal, Québec Canada

**Keywords:** Child, Parent, Symptoms of depression, Neighborhood, Safety

## Abstract

**Background:**

Physical characteristics and perceptions of an environment can have enduring effects on one’s mental health. The present study aimed to determine whether a set of measures of neighborhood safety – independent built environment features, parents’ perception of neighborhood safety and community cohesion, and children’s perception of neighborhood safety – best related to symptoms of depression in young boys and girls from Montréal, Québec.

**Methods:**

Data were from the Quebec Adipose and Lifestyle Investigation in Youth (QUALITY) cohort. Participants were aged 8 to 10 years (M = 9.5) at baseline (2005). Measures included: child symptoms of depression, neighborhood perception (child and parentally reported), and independent rater assessed visible disorder/neighborhood safety issues and road safety features. Multiple linear regressions were used to examine the relation between neighborhood safety measures and symptoms of depression for boys (*n* = 262) and girls (*n* = 212) at baseline and at follow-up time approximately 2 years later in 2008 after adjusting for baseline sex, age, body mass index, pubertal status, physical activity, family income, parent education, population density, and land-use mix. Analyses were sex stratified.

**Results:**

Greater child perceived safety was associated with lower symptoms of depression in boys at both baseline and follow-up, and greater community cohesion was associated with lower symptoms of depression in girls at baseline. These results were not maintained after adjusting for multiple testing.

**Conclusion:**

Future research should elucidate the more precise pathways linking neighborhood safety to symptoms of depression among children and consider differences across sexes.

## Background

The prevalence of mental health disorders and hospitalizations due to related mental health concerns continues to increase in Canadian children and adolescents [1]. Symptoms of depression are a common mental health concern that is typically associated with a short period of time, and do not necessarily impair daily functioning significantly. It refers to an emotional state of feeling sad and can be an early indicator of clinical depression [2]. In contrast, having depression or major depressive disorder (MDD) refers to a broader term that encompasses a range of persistent symptoms and is a clinical diagnosis characterized by a persistent and intense feeling of sadness or a lack of interest in activities [3]. It is a mental health disorder that can significantly affect daily life. Research indicates that the early stages of symptoms of depression substantially increase one’s chance of developing MDD [4].

Symptoms of depression have been related to several factors, one of them being feeling unsafe. In particular, feeling unsafe in one’s residential neighbourhood has been associated with reduced sleep quality [2] which increases one’s risk for mental health concerns (e.g., depression and anxiety) among children [5]. Unsafe neighborhood environments have also been shown to lead individuals to be less likely to spend time outdoors or less likely to benefit from social interactions, which have been associated with poorer physical and mental health outcomes (e.g., increased body mass index (BMI), increased anxiety, and increased symptoms of depression) [6, 7].

Research suggests that both objective measures and subjective perceptions are equally relevant in shaping individuals’ feelings of neighborhood safety [8]. Among objective measures, the presence of vacant lots and high traffic levels have been negatively associated with feelings of safety [9], while the presence of sidewalks and crossing guards has been positively associated [10]. Moreover, subjective measures, such as perceived low community cohesion and perceived low neighborhood ‘curb appeal’, have been associated with reduced feelings of safety in one’s community [8]. In addition, our previous research has shown that children’s perceptions of their neighborhood safety differ from those of their parents [9]. We have found that children tend to draw inferences of safety from physical features (e.g., the nature of the environment including number of trees and street lights) while adults rely more heavily on sociocultural cues (e.g., neglect of the environment such as disorder and poorly maintained buildings) [9]. For parents’ perceptions, safety is related not only to physical cues, but also to social cues, such as community cohesion and community involvement [11]. For instance, a built environment with social interaction features (e.g., buildings with porches) led to reduced symptoms of depression due to increased perceived community cohesion and involvement [12].

Despite the evidence of how neighborhoods can impact one’s health, particularly their mental health, to date, there have been a limited number of studies on neighborhood characteristics and symptoms of depression in children. Further, studies often examine the built environment by combining objective and subjective perceptions of safety, preventing these distinct measures from being disentangled and analyzed separately. Studies have also only looked at one perspective, neglecting the fact that perceptions differ across parent and child. In particular, because children’s experience of the visual world differs from that of adults [13], incorporating children’s perceptions is imperative to understanding how features of an environment impacts children’s mental health. Additionally, there are even fewer studies that have analyzed sex differences in a child sample, despite research showing that sex is a factor that contributes to neighborhood perceptions in adults [14] and that sex differences have been found in varying levels of symptoms of depression in children [15].

To address these gaps in the literature, this study aims to determine whether objective measures, or subjective perceptions (parents’ perception of neighborhood safety, parents’ perception of neighborhood community cohesion, and children’s perception of neighborhood safety) best predict symptoms of depression in a large cohort of boys and girls at two time points (baseline and at follow-up). By addressing these gaps, this study provides a nuanced understanding of how neighborhood environments are associated with children’s mental health, considering both subjective and objective measures and accounting for sex-specific differences. This approach can help target effective interventions aimed at improving mental health outcomes for children in diverse neighborhood contexts, much like the surrounding boroughs in Montréal, Québec, the setting of the current study [16].

## Method

### Data

Data were drawn from the Quebec Adipose and Lifestyle Investigation in Youth (QUALITY) study, an ongoing longitudinal cohort of the natural history of obesity and cardiovascular risk of children living in the province of Québec (*n* = 630). Details on cohort eligibility and selection can be found elsewhere [17]. Briefly, families were recruited using a school-based sampling strategy. Eligible participants consisted of Caucasian children aged 8–10 years with at least one biological parent with obesity. Parents completed in-person self-administered questionnaires while children completed interviewer-administered questionnaires. Baseline data when children were aged 8–10 years were collected between September 2005 and December 2008. Follow-up data when children were aged 10–12 years were collected between September 2008 and March 2011 [17]. Written informed consent was obtained from parents, and assent was provided by the children prior to beginning the study. The study received approval from the Ethics Board of the CHU Sainte-Justine University Hospital Center and the Institut Universitaire de Cardiologie et de Pneumologie du Québec at Université Laval. This secondary data analysis was approved by the Concordia University Research Ethics Board (#30013531).

### Measures

Objective and subjective measures of the neighborhood (Table 1) were assessed at baseline, following the guidelines and recommendations in the literature [9]. Objective measures included the presence of visible disorder/neighborhood safety issues, and the presence of road safety features. Subjective measures included the perceived neighborhood safety (parent and child reported), as well as the perceived neighborhood community cohesion (parent reported) as further described below.

**Table 1:** Dimensions of perceived neighborhood safety

Objective	Subjective
Indicators of neighborhood disorder (e.g., litter, graffiti, poor lighting)	Perceived neighborhood community cohesion (parent reported)
Indicators of pedestrian road safety (e.g., stop signs, speed bumps, cross walks)	Perceived neighborhood safety (parent and child reported)

#### Objective measures of the neighborhood

##### Neighborhood features

At baseline, neighborhood features were measured by adopting an ego-centered approach that considered the surrounding environment of the family’s residence [18]. These data were only available for those living in the Greater Montréal area; the procedure has been previously published and is briefly explained here [9, 18]. A pair of trained observers independently audited up to ten street segments surrounding the residence by recording features of the physical microenvironment using an adapted version of a neighborhood assessment tool [19]. These street segments represent roughly a 200-meter to 400-meter area surrounding the participants’ residential addresses.

Montréal is known for its cultural richness, bilingualism, and diversity. The greater Montréal area is the most populous metropolitan area in the province of Québec, Canada. Around the time of the study, it was identified as Canada’s most unequal and most segregated major city regarding income [20]. In 2006, the population exceeded one million, with a density ranging from 12,000 inhabitants/km² in central boroughs to 5,000 people/km² in suburban neighborhoods [21].

For the purpose of this study, neighborhood features were divided into two broader categories: visible disorder/neighborhood safety issues, and road safety features. Detailed information on the neighborhood features can be found elsewhere [22]. Visible disorder/neighborhood safety issues consisted of 5 items which assessed the presence of graffiti, garbage, broken or abandoned items and appliances, vandalized buildings, and insufficient lighting. Road safety features consisted of 17 items that assessed the presence of various traffic calming measures (e.g., stop signs, speed bumps, cross walks, speed limits, school zone signs). As previously documented in our work [9, 22], scores for these two measures were calculated as whether or not at least one street segment noted the presence of visible disorder/neighborhood safety concern or road safety features.

#### Subjective measures of the neighborhood

At baseline, parents (mother and father together in a single session) and children (separately from their parents) completed questionnaires pertaining to their neighborhood, as described in further detail in this section.

##### Child perceived safety

As defined in our previous research [9], a global measure of children’s feelings of safety was conceptualized as measures of perceived safety to walk in one’s neighborhood. At baseline, children’s perceived neighborhood safety was assessed with four items: “There is no danger walking around or cycling alone in my neighborhood [during the day],” and separately, “[at night]”, “It is safe to bike or walk outside due to traffic”, and “Other children my age plays outside”. Scores were on a four-point Likert scale that ranged from 0 (completely disagree) to 3 (completely agree) such that greater scores indicated greater child perceived safety. These variables all correlated positively (*r* = 0.5, *p* < 0.01) with one another. A total score was calculated by taking the mean as recommended in the literature [9].

##### Parent perceived safety

At baseline, parents’ perceived neighborhood safety was assessed with three items: the neighborhood “is at high risk for crime,” “is attractive,” and “kids can play outside without danger”. Similar measures have been used in the literature to assess this construct [23]. Scores were on a five-point Likert scale that ranged from 0 (not true at all) to 4 (very true). Items were reverse coded as needed such that greater scores indicated greater parent perceived safety. A total score was calculated by taking the mean of the items [9].

##### Parent perceived neighborhood cohesion

At baseline, parents’ perceived neighborhood cohesion was assessed with the following items: “When there is a problem, neighbors come together to solve it,” “In our area, some adults are role models,” “People in this area are ready to help neighbors,” and “We can trust adults in this area to make sure children are safe”. Responses were on a four-point Likert scale ranging from 0 (completely disagree) to 3 (completely agree). Items were reverse-coded as needed so that greater mean scores indicated greater parent perceived neighborhood cohesion [9].

##### Symptoms of depression

At both baseline and follow-up, children responded to the 12-item Centre for Epidemiological Studies-Depression Scale (CES-D-12) from the National Longitudinal Study of Children and Youth [24]. The scale assesses the frequency of experiencing depressive symptoms within the past week (e.g., “I felt lonely”) on a four-point Likert scale ranging from 0 (none of the time) to 3 (most or all of the time). As recommended in the literature [25], three reverse-coded positively worded items (e.g., “I enjoy life”) were removed as they have been shown to reduce the internal consistency of the scale. The responses from the nine remaining items were subsequently summed for a total score (range: 0–27), with higher scores representing higher levels of depressive symptoms (Cronbach’s alpha at baseline and follow-up: 0.65–0.70). According to the CES-D-12, a score of 9 or lower generally suggests a lower likelihood of experiencing significant depressive symptoms, indicating minimal depressive symptoms [19].

#### Covariates

Covariates considered for inclusion in the analyses were those identified as related to symptoms of depression in the literature: BMI [26], pubertal status [27], children’s physical activity levels [5], as well as general land use of the neighborhood [28], and sociodemographic characteristics of the household. These are explained in further detail, below.

##### BMI

Children’s height and weight were measured by trained study staff at baseline using standard protocols. These values were used to calculate BMI, which were then compared to the World Health Organization reference curves to calculate BMI z-scores to account for the child’s sex and age [29].

##### Pubertal status

Pubertal status (sexual maturation) of the child at baseline was scored by trained nurses according to Tanner stages [27].

##### Physical activity

Physical activity was assessed at baseline with accelerometer data and categorized as moderate (3201–8200 steps/day) and vigorous (> 8200 steps/day) daily activity in accordance to the literature and summed together [13]. We then created a dichotomous variable indicating whether the child met the guideline of at least 60 min of activity a day [5].

##### Household characteristics

Household characteristics of interest included household income and parental education (both assessed at baseline). Parent education was recoded into a dichotomous variable on whether at least one parent had a university degree or higher. Based on published work utilizing our dataset and the income distribution within our sample, household income was also recoded into a dichotomous variable, indicating whether the total household income in the preceding year (before taxes and deductions) was at least 50,000 CAD [17, 30].

##### Neighborhood characteristics

Land use mix is an indicator of neighborhood heterogeneity, where a higher score indicates greater heterogeneity such that a residential area also has offices, retail, entertainment, and other services nearby. Population density was kept as a continuous variable while land use mix was computed using an entropy index that measured the homogeneity or diversity of land use within a neighbourhood. These measures were based on CanMap (DMTI Spatial Inc., Markham, ON, Canada) information using a 500 m network buffer centered on the household residence. Land use mix was dichotomized into low land use mix (lowest 2 tertiles) and high land use mix (upper tertile) as described in a previous study [31].

### Statistical analysis

To determine our analytical sample size, we retained all participants with neighborhood audit data (excluding 118 participants), and who had not moved between baseline and follow-up (excluding 38) resulting in a sample size of 474. School-based clustering did not need to be accounted for as very few participants were recruited from the same schools [32]. Data missingness was negligible with counts of 5 or less for all variables except symptoms of depression at follow-up (11% missing) and minutes of physical activity (17% missing). Multiple imputation using Markov Chain Monte Carlo (*n* = 50 imputed datasets) was used to address data missingness on the variables of interest. Auxillary variables included all variables of interest as well as sociodemographic characteristics. For the imputed dataset, estimates were pooled as recommended in the literature [33].

Symptoms of depression was not normally distributed in this dataset. However, as results were consistent in direction, magnitude, and conclusions with (log, squared, cubed) or without transformation, we made the analytic decision to present only untransformed results to maximize interpretability. First, sex stratified descriptive statistics were produced, and differences between boys and girls were assessed using t-tests and chi-square tests. Multiple linear regressions to examine the relation of neighborhood safety features and subjective perceptions with symptoms of depression at baseline and at the two-year follow-up were conducted. Regression models assessed symptoms of depression scores at (1) baseline and (2) at follow-up based on baseline measures of neighborhood safety. All analyses adjusted for covariates at baseline: age, BMI z-scores, pubertal status, whether the child met physical activity recommendations, family income, parent education, population density, and land use mix. We tested for multicollinearity in the multiple linear regressions with the variance inflation factor.

A sensitivity analysis was also conducted in which a parsimonious model containing only the neighborhood features, perceived neighborhood features, age, and BMI z-score were tested. Lastly, to correct for multiple testing, the Benjamini-Hochberg method was used on all the measures across both times and sexes (i.e., 20 variables) [34]. Specifically, the *p*-values for the primary measures of interest were ranked in increasing order based on their magnitude, and compared to the adjusted alpha level (defined as: (i/m)*Q where i = rank, m = total number of tests, and Q = 0.05) as recommended in the literature [34]. All analyses were conducted with SAS (9.4).

## Results

The analytic sample did not differ from the full cohort in the following baseline characteristics: age, sex, BMI z-score, highest parental education, symptoms of depression, and parent or child perceptions of the neighbourhood. However, those excluded were more likely to have a baseline household income of less than $50,000 CAD/year. In the analytic sample, the mean age of participants was 9.59 years for males and 9.61 years for females at baseline with approximately 55% being a boy (Table 2). The average length of time between baseline and follow-up was 2 years (25 months) and ranged from 23 to 36 months.

Demographic characteristics were similar between boys and girls with regards to parent education and household income (*p* > 0.05). A higher portion of girls started puberty at baseline compared to the boys (39.6% vs. 8.8%, respectively; *p* < 0.0001) and also had a lower BMI z-score, although this was not statistically significant (0.99 vs. 1.08, respectively; *p* = 0.12). Additionally, boys were more likely to meet physical activity recommendations than girls (13.4% vs. 2.8%; *p* < 0.0001). No sex differences in symptoms of depression levels at baseline or follow-up were detected, nor in any of the safety measures (objective and perceived).

In the studied sample, individual total scores reflecting symptoms of depression were generally low at both baseline (boys mean = 3.49, girls mean = 3.77, *p* = 0.22) and follow-up (boys mean = 3.54, girls mean = 3.67, *p* = 0.19).

**Table 2:** Characteristics of the population (*n* = 474) at baseline and follow-up from pooled estimates across 50 multiple imputed datasets

Characteristics^1^	Males (*n* = 262)	Females (*n* = 212)	*p*
Age in months, mean (SD)	115.05 (10.8)	115.37 (11.1)	0.16
Body mass index z-score (BMI z-score)	1.08 (1.3)	0.99 (1.3)	0.12
Parent(s) with university degree, %	54.2%	53.8%	0.93
Household income < $50,000, %	63.0%	64.6%	0.75
In puberty, %	8.8%	39.6%	< 0.0001
Meets guidelines for daily PA (*≥* 60 min), %	13.4%	2.8%	< 0.0001
Symptoms of depression, mean (SD)			
Baseline	3.49 (2.9)	3.77 (3.2)	0.22
Follow-up	3.54 (2.9)	3.67 (3.4)	0.19
Perception safety measures, mean (SD)			
Child perceived safety	1.47 (0.5)	1.38 (0.5)	0.13
Parent perceived safety	3.24 (0.7)	3.23 (0.7)	0.16
Parent perceived community cohesion	2.13 (0.6)	2.13 (0.6)	0.16
Objective safety measures, %			
At least one visible disorder/neighborhood safety issue feature	37.4%	30.7%	0.12
At least one road safety feature	68.7%	70.8%	0.63
High land-use mix (top and middle tertile), %	33.2%	34.0%	0.93
Population density per Km^2^	3740.6 (3032.4)	3249.3 (2603.9)	0.09

^1^all values presented are at baseline unless otherwise specified; missing data that were imputed include: symptoms of depression at baseline (*n* = 2), symptoms of depression at follow-up (*n* = 52), Tanner stage (*n* = 1), household income (*n* = 4), highest parental education (*n* = 3), physical activity (*n* = 82); age, BMI z-score, sex, subjective neighborhood measures and objective neighborhood measures had no missing data

Symptoms of depression at baseline and follow-up were moderately correlated with one another (*r* = 0.32, *p* < 0.0001, Table 3). Symptoms of depression at both baseline and follow-up were significantly negatively correlated to child perceived safety (*r*=-0.11, *p* < 0.05 and *r*=-0.12, *p* < 0.01 respectively). Parent and child perceptions of safety were positively but weakly correlated with one another (*r* = 0.13, *p* < 0.01). Visible disorder/neighborhood safety issues was moderately negatively correlated with parent perceived safety (*r*=-0.36, *p* < 0.0001) and positively correlated with road safety (*r* = 0.27, *p* < 0.0001). Because all the different safety measures were not strongly correlated with one another and variance of inflation was low (e.g., all below 1.4), evidence of multicollinearity was low. Accordingly, all the safety measures were included in the regression models simultaneously.

**Table 3:** Pearson correlation coefficient’s (r) between symptoms of depression and safety measures from pooled estimates across 50 multiple imputed datasets (*n* = 474)

	Symptoms of depression Baseline (1)	Symptoms of depression follow-up (2)	Child perceived safety (3)	Parent perceived safety (4)	Parent perceived community cohesion (5)	Visible disorder/neighbor-hood safety issues (6)	Road safety features (7)
1							
2	0.32***						
3	-0.11*	-0.12**					
4	-0.09*	-0.04	0.13**				
5	-0.05	-0.10*	0.09	0.47***			
6	0.007	-0.05	0.06	-0.36***	-0.20***		
7	0.03	0.007	0.02	-0.03	-0.08	0.27***	

* <0.05; ** <0.01; *** <0.0001

### Multiple linear regression: at baseline

Greater child perceived safety was associated with lower symptoms of depression, but only in boys (Table 4). For instance, the more boys perceived their neighborhood to be safe (feeling safe walking, cycling, and playing outside), the lower symptoms of depression they reported (B=-1.08, 95% Confidence Interval [CI]: -1.79, -0.37). However, this relation was not found in girls (B = 0.03; 95% CI: -0.80, 0.87). In contrast, greater parent perceived neighborhood cohesion (the level of neighborhood trust and support) was associated with lower symptoms of depression in girls (B=-0.81, 95% CI: -1.60, -0.01), but not in boys. Objective measures of neighborhood safety (visible disorder/neighborhood safety issues, road safety features) and parent perceived safety were not associated with boy’s or girl’s symptoms of depression. When correcting for multiple testing using the Benjamini-Hochberg method [34], the parent perceived neighborhood cohesion association found in girls was no longer observed (*p*-value: 0.047; adjusted significance level: 0.0075); the child perceived safety association found in boys was also no longer observed (*p*-value: 0.0029; adjusted significance level: 0.0025).

### Multiple linear regression: at follow-up

Results were relatively consistent at follow-up. Greater child perceived safety at baseline remained associated with lower symptoms of depression at follow-up among boys (B=-1.06, 95% Confidence Interval [CI]: -1.81, -0.32), but not for girls. Results of the sensitivity analysis were consistent with our main findings, with the exception that higher community cohesion was now also significantly associated with lower symptoms of depression at follow-up among the girls. However, community cohesion was no longer significant when correcting for multiple testing using the Benjamini-Hochberg method [34], and child perceived safety with depression at follow-up among boys was only marginally significant (*p*-value: 0.005; adjusted significance level: 0.005).

**Table 4:** Pooled estimates from multiple linear regressions (*n* = 50 multiply imputed datasets) stratified sample by sex

	Baseline (2005)				Follow-up (2008)			
	Males (*n* = 262)		Females (*n* = 212)		Males (*n* = 262)		Females (*n* = 212)	
	B (SE)^a^	95% CI	B (SE)^a^	95% CI	B (SE)^a^	95% CI	B (SE)^a^	95% CI
*≥* 1 visible disorder/neighborhood safety issue feature	-0.04 (0.5)	-0.99, 0.91	-0.08 (0.6)	-1.28, 1.11	-0.57 (0.5)	-1.53, 0.39	-0.75 (0.7)	-2.12, 0.62
*≥* 1 road safety feature	0.10 (0.4)	-0.75, 0.94	0.42 (0.5)	-0.58, 1.41	0.23 (0.4)	-0.62, 1.08	-0.23 (0.6)	-1.38, 0.92
Perceived safety (child)	-1.08 (0.4)**	-1.79, -0.37	0.03 (0.4)	-0.80, 0.87	-1.06 (0.4)**	-1.81, -0.32	0.07 (0.5)	-0.85, 0.99
Perceived safety (parent)	-0.24 (0.4)	-0.98, 0.49	-0.55 (0.4)	-1.29, 0.19	-0.18 (0.4)	-0.96, 0.60	0.007 (0.5)	-0.88, 0.89
Community cohesion (parent)	0.74 (0.4)	-0.05, 1.54	-0.81 (0.4)*	-1.60, -0.01	0.18 (0.4)	-0.65, 1.00	-0.87 (0.5)	-1.81, 0.07
Age (in months)	0.02 (0.02)	-0.02, 0.05	-0.04 (0.02)	-0.09, 0.003	-0.03 (0.02)	-0.06, 0.01	-0.01 (0.03)	-0.06, 0.04
BMI-Z	0.10 (0.1)	-0.18, 0.38	0.04 (0.2)	-0.29, 0.38	-0.04 (0.2)	-0.32, 0.25	0.23 (0.2)	-0.16, 0.63
Pubertal	0.09 (0.7)	-1.25, 1.43	0.97 (0.6)	-0.14, 2.07	0.16 (0.7)	-1.18, 1.50	-0.22 (0.6)	-1.45, 1.00
*≥* 1 parent with uni. education	-0.05 (0.4)	-0.87, 0.77	-0.10 (0.5)	-1.01, 0.82	-0.46 (0.4)	-1.32, 0.40	-1.14 (0.5)*	-2.19, -0.10
Household income < 50 K/year	0.55 (0.4)	-0.27, 1.37	-0.07 (0.5)	-1.03, 0.88	0.09 (0.4)	-0.73, 0.91	0.03 (0.5)	-1.02, 1.09
Population density per100K	-0.84 (8.5)	-17.4, 15.7	-12.31 (10.8)	-33.6, 8.9	10.82 (8.6)	-6.0, 27.7	8.21 (12.4)	-16.1, 32.6
High land use mix^b^	0.13 (0.5)	-0.86, 1.12	0.11 (0.6)	-0.97 1.19	0.02 (0.5)	-1.02, 1.05	-0.47 (0.6)	-1.71, 0.77
Meets PA guidelines^c^	0.36 (0.6)	-0.81, 1.52	0.17 (1.3)	-2.47, 2.80	0.36 (0.6)	-0.88, 1.60	0.24 (1.6)	-2.96, 3.44

PA: physical activity; * *p* < 0.05; ** *p* < 0.01; *** *p* < 0.0001 prior to accounting for multiple comparisons

^a^Estimates are adjusted for all variables shown here; ^b^Top and middle tertile compared to lowest tertile; ^c^Whether the child met the guideline of 60 min of activity a day or not

## Discussion

This study assessed the association between a total of five indicators (2 objective, 3 subjective) of neighborhood safety and symptoms of depression among boys and girls at baseline and at two-year follow-up. Regression models assessed symptoms of depression scores at both time points across the sexes on measures of neighborhood safety. Analyses were also adjusted for multiple testing using the Benjamini-Hochberg method [34].

Higher child perceived safety in boys was the only safety measure that was associated with lower symptoms of depression at both baseline and follow-up. However, these results were at best, marginally significant after correcting for multiple comparisons. In line with previous research, a possible association exists between self-perceptions and male children’s mental health [5]. Further research is warranted to determine the strength of this relationship.

Moreover, previous studies show that various environmental features (e.g., trees, lighting, traffic, and visible minorities) differently predict parents’ and children’s perceived neighborhood safety [9]. Studies such as ours demonstrate that child and parent perceptions of safety are weakly correlated. This may suggest that parents and children form their separate perceptions of safety. Additionally, subjective perceptions were weakly correlated with objectively measured features. While the literature suggests that objective measures may capture important structural aspects of the environment that a resident inhabits, subjective neighborhood measures assess various aspects of a neighborhood, capturing how satisfied young people are with their environment and how safe they feel [35]. Results underscore the importance of measuring various safety-related aspects, as they can reflect different dimensions of neighborhood perception and can have distinct associations with symptoms of depression.

Additional notable sex differences were detected. For instance, while the literature has shown that adult women tend to have lower levels of perceived personal safety than men [36], we did not see these sex differences in a youth population. Our data demonstrated that girls and boys reported similar levels of perceived neighborhood safety at ages 8–10 years. In addition, greater parent perceived neighborhood cohesion was associated with lower symptoms of depression in girls at baseline. Consistent with previous research, girls may benefit more from cohesive neighborhood environment characteristics more than boys, as found in a 2008 meta-analysis [37]. The authors of this meta-analysis revealed that mixed sex results were observed when studying neighborhood characteristics on symptoms of depression with adolescent girls having lower levels of symptoms of depression when they felt safer and lived in a greater cohesive neighborhood. Another study reported that in neighborhoods with high violence, low levels of parent-to-parent conflict buffered girls from developing depressive symptoms, but not for boys [38]. However, in our study, this relationship between parent perceived neighborhood cohesion and symptoms of depression was no longer significant for girls after adjusting for multiple comparisons.

Although study results are not significant after controlling for multiple comparisons, caution is warranted when interpreting the findings both before and after this adjustment. While running multiple tests increases the risk of Type I errors, adjustments for multiple comparisons can also raise the likelihood of Type II errors, potentially obscuring meaningful associations in the data [39]. The results of this study should be interpreted with the following additional limitations in mind. First, this study is unique in that it draws from a sample of children with at least one parent with obesity and of Western European descent in Montréal, Québec. Thus, while the prevalence of obesity among adults of child-bearing age in Canada has been increasing steadily over the past several years, the study’s results may not be representative of the general Québec population [17]. We used multiple imputations to account for any data missingness due to the 10% lost to follow-up. Although imputing for dependent variables (symptoms of depression in this study) historically was cautioned against, recent simulation and methodological studies suggest that this is a misconception [40]. We were unable to assess the likelihood of having significant depressive symptoms (scores *≥* 10) given the relatively low averages in this sample. Investigating these associations in a sample with higher depressive symptom scores is needed. Moreover, neighborhood safety measures were only available at baseline. It is possible that this led to measurement error if indicators or perceptions of neighborhood safety changed during this time frame. Finally, a large number of comparisons were made in this study, raising the potential for inflated Type I errors. To address this, we applied corrections for multiple comparisons using the Benjamini-Hochberg method as recommended in the literature [34]. However, as mentioned, these corections may have inflated Type II errors.

Considering the findings of the current study alongside the similar and distinct results from previous research, further research is necessary to gain a better understanding of the mechanisms affecting safety in the environments of both girls and boys, and how these factors impact their mental health.

## Conclusion

Out of all five safety indicators assessed (child perceived safety, parent perceived safety, parent perceived community cohesion, objectively measured visible disorder/neighborhood safety issues, objectively measured pedestrian reported road safety), child perceived safety was the only significant measure related to symptoms of depression at both baseline and follow-up in boys from Montréal, Canada. We did not find any association between child perceived safety and symptoms of depression in girls. However, parent-reported community cohesion was significantly related to symptoms of depression at baseline for girls. These may have been spurious findings given the number of independent measures being tested as neither was no longer significant after adjusting for multiple comparisons. Research is needed to further investigate this. The results serve as a cautionary reminder of a potential relationship between children’s perceptions of safety and their mental health, with differences observed between boys and girls. Future directions should continue to explore the relations among both objective and subjective neighborhood features in the two sexes. Additionally, public policy should work towards ensuring adequate safety measures are maintained in neighborhoods to ensure optimal mental health of children.

## Data Availability

All data that support the findings of this study are available from the QUALITY research team upon reasonable request.
